# Household wealth and timing of first birth among highly educated women in sub-Saharan Africa: evidence from survey-adjusted survival analysis

**DOI:** 10.3389/frph.2026.1805036

**Published:** 2026-05-14

**Authors:** Sylvia David Kachola, Redson Mwandama, Gladson Andrew Chipala, Margubur Rahaman, Hannah Dunga

**Affiliations:** 1 The French Institute for Demographic Studies (INED), European Doctoral School of Demography, Aubervilliers, France; 2 Centre for Social Research, University of Malawi, Zomba, Malawi; 3 School of Law, Economics, and Government, Department of Economics, University of Malawi, Zomba, Malawi; 4 Research Consultant, Govind Ballabh Pant Social Science Institute, Prayagraj, India; 5 Department of Economics, University of South Africa Pretoria, South Africa

**Keywords:** female education, fertility timing, household wealth, sub-Saharan Africa, survival analysis

## Abstract

**Introduction:**

Female education is widely recognized as an important factor associated with fertility decline, yet fertility transitions in sub-Saharan Africa remain uneven. This study examines whether household wealth is associated with the timing of first birth among women who have already attained higher education.

**Methods:**

The analysis uses recent Demographic and Health Surveys from thirty-four countries (2010-2024) and a pooled sample of 17,336 highly educated women. Survey-adjusted Kaplan-Meier estimates and proportional hazards models (Cox, Weibull, Gompertz) are applied to assess socioeconomic gradients in first-birth timing. Regional patterns are compared across Southern, Eastern, Western, and Central Africa.

**Results:**

The survival curves reveal clear socioeconomic gradients: by age 25, more than half of women in the poorest quintile have had a first birth, compared with only about one third of those in the richest quintile. Cox, Weibull, and Gompertz models consistently show that women in lower wealth quintiles have higher hazards of first birth (19% higher for richer, 28% for middle, 42% for poorer, and 33% for the poorest women relative to the richest). These patterns remain robust across model specifications. Regional analyses show that the gradient is strongest in Southern Africa, moderate in East Africa, and more compressed in West and Central Africa.

**Discussion:**

The findings indicate that even among highly educated women, household wealth remains strongly associated with fertility timing, independently of formal schooling. The study extends the 'diverging destinies' framework by showing that socioeconomic stratification persists within highly educated groups, underscoring the relevance of wealth inequality and economic insecurity alongside educational expansion in shaping fertility patterns.

## 1 Background

Sub-Saharan Africa remains the global hotspot of high fertility. Although the region's total fertility rate (TFR) declined from approximately 6.5 to 4.3 between 1980 and 2024, the pace of fertility decline has been slower than in other regions of the world, raising persistent socio-demographic and public health concerns (1). At the same time, substantial spatial inequalities in fertility persist across and within countries, reflecting deep-seated heterogeneity in socioeconomic development, institutional capacity, and access to reproductive health services (2). Understanding the mechanisms underlying these spatial and socio-demographic disparities in fertility remains a critical research and policy priority, particularly in the context of achieving the Sustainable Development Goals (SDGs) related to health, gender equality, and poverty reduction in sub-Saharan Africa (3).

The theoretical background of the proximate determinants of fertility strongly recognizes female education as a key social instrument of fertility decline (4). Based on existing fertility theory and empirical evidence, specifically the proximate determinants framework (5) and demographic transition theory, studies find that women's education is associated with fertility not only at the individual level but also at the community level, as educated women tend to exhibit different norms and behaviors compared to the less educated (6). As societies transition toward higher levels of female educational attainment, fertility behavior tends to shift away from high-risk patterns toward delayed and more deliberate fertility, facilitated by increased use of modern contraception, greater female labor-force participation, and strengthened realization of sexual and reproductive rights (7, 8). However, while education has progressed markedly, it is important to note that the relationship between education and fertility is not strictly one-directional: although higher education generally reduces fertility, reduced childbearing may in turn enable women to pursue more schooling, pointing to a bidirectional association (9). The mechanisms through which education affects fertility are therefore complex, and attributing fertility change solely to education without considering broader socioeconomic factors risks oversimplification (10, 11). Across sub-Saharan Africa over recent decades, the expected fertility declines have not always materialized uniformly, suggesting that education's effects may be moderated by other socioeconomic factors (12).

Emerging scholarship points to growing socioeconomic stratification in fertility timing across sub-Saharan Africa, with evidence of “diverging destinies” in family formation patterns (12). Women with high levels of education and economic resources increasingly delay marriage and first birth to their late twenties or early thirties, while their less advantaged counterparts continue to enter motherhood in their late teens and early twenties (13). This divergence has been documented in countries experiencing rising economic inequality, where wealth appears to amplify the effects of education on reproductive timing (14). Yet a critical research question remains unanswered using recent nationally representative demographic data in sub-Saharan Africa: among women who have already attained higher education, does household wealth continue to predict the timing of first birth?.

This question is theoretically important because it challenges the conventional wisdom that education alone predicts fertility timing (15). If wealth gradients persist even among highly educated women, this implies that economic resources are independently associated with fertility timing beyond human capital. These pathways include higher opportunity costs of early childbearing, greater access to high-quality employment, stronger exposure to norms that favour delayed fertility, and enhanced ability to implement fertility preferences through effective contraception. It is worth noting that many of these pathways overlap substantially with those attributed to education, since education and household wealth are themselves highly correlated in sub-Saharan Africa (16, 17). Wealthier households invest more in children's schooling, and more educated women tend to access better-paying employment. Disentangling the independent contributions of education and wealth is therefore inherently difficult in cross-sectional data, and the pathways described here should be understood as jointly operating rather than entirely separate mechanisms (18). Understanding these dynamics among educated elites is particularly relevant for policy, as this group represents the future leadership and economic drivers of sub-Saharan African societies.

Recent evidence complicates the traditional opportunity-cost framework of fertility. Experimental and quasi-experimental studies from several sub-Saharan African countries show that interventions increasing women's income and wealth can, counterintuitively, raise fertility, particularly among women without sons (19). These findings suggest that childbearing may function as a strategy for securing economic security in later life in contexts where formal pension systems and social protection remain largely absent (20, 21). Such evidence underscores the importance of institutional and normative contexts, including inheritance regimes, marriage systems, and the availability of social protection, in shaping how economic resources translate into reproductive decisions. In the present study, these contextual factors are not directly measured but are partially accounted for through country fixed effects in the regression models, which absorb unobserved national-level differences in institutional arrangements and normative environments. This approach cannot fully isolate the mechanisms through which context operates, and this remains an important limitation acknowledged in the limitations section.

Motivated by this emerging gap in the literature, the present study is designed to examine the interaction between education, economic resources, and fertility timing. The study contributes to the literature in three key ways. First, by focusing exclusively on women who have attained higher education, it isolates the independent association of household wealth from confounding educational gradients, allowing for a cleaner test of economic stratification within an elite subpopulation. Second, by employing survival analysis techniques, the study examines the timing and pace of entry into motherhood across wealth quintiles, offering dynamic insights into how economic resources correlate with life-course transitions among highly educated women. Third, using survey-adjusted hazard models, the analysis estimates wealth differentials in the risk of first birth and assesses regional heterogeneity across West, East, and Southern Africa. In doing so, the study extends the diverging destinies framework by demonstrating that socioeconomic stratification persists even within highly educated populations. This builds on a growing body of work showing that socioeconomic gradients can diverge within high-status subgroups in ways that are invisible at the population level, an insight analogous to the ecological paradox, where population-level associations can mask or even reverse within-subgroup patterns (12, 18).

## 2 Methodology

### 2.1 Data

This study uses the most recent Demographic and Health Surveys from thirty-four Sub-Saharan African countries (see Table 1). These surveys cover West Africa, East Africa, Southern Africa, and Central Africa. The DHS program applies a standardized two-stage stratified cluster sampling design that ensures national representativeness across countries and survey years. Individual women's datasets from each country were downloaded and appended to create a harmonized pooled dataset suitable for cross-national analysis. The regional estimates presented in this study should be interpreted as pooled summaries of the countries included in the analytic sample, rather than fully representative of all countries in each region. Some countries contribute a larger share of highly educated women than others, which means regional patterns reflect the composition of the pooled sample.

**Table 1:** Study sample descriptions from recent demographic and health surveys (34 countries).

Country Name	DHS Code	Region	Year(s)	Total N	Higher Ed. (%)
Angola	AO8	Southern Africa	2023/24	10,466	5.36%
Lesotho	LS8	Southern Africa	2023/24	4,446	16.10%
Malawi	MW8	Southern Africa	2024	16,609	2.07%
Mozambique	MZ8	Southern Africa	2022/23	9,956	2.16%
Namibia	NM6	Southern Africa	2013	6,453	7.92%
South Africa	ZA7	Southern Africa	2016	6,115	11.89%
Zambia	ZM8	Southern Africa	2024	10,227	7.01%
Zimbabwe	ZW6	Southern Africa	2010/11	6,725	4.15%
Burundi	BU7	East Africa	2016/17	11,081	0.80%
Ethiopia	ET7	East Africa	2011	5,846	3.50%
Kenya	KE8	East Africa	2022	23,343	17.61%
Comoros	KM6	East Africa	2012	2,934	5.84%
Rwanda	RW7	East Africa	2019/20	9,214	4.11%
Tanzania	TZ8	East Africa	2022	11,116	1.09%
Uganda	UG7	East Africa	2016	13,744	7.00%
Benin	BJ7	West Africa	2017/18	11,756	1.35%
Burkina Faso	BF8	West Africa	2021	12,931	1.51%
Côte d’Ivoire	CI8	West Africa	2021	10,942	4.11%
Ghana	GH8	West Africa	2022/23	10,356	8.17%
Gambia	GM7	West Africa	2019/20	7,924	5.29%
Guinea	GN7	West Africa	2018	7,802	2.48%
Liberia	LB7	West Africa	2019/20	6,408	5.44%
Mali	ML8	West Africa	2023/24	12,775	2.15%
Mauritania	MR7	West Africa	2019/20/21	10,037	1.62%
Nigeria	NG8	West Africa	2023/24	26,397	12.15%
Niger	NI6	West Africa	2012	9,197	0.40%
Sierra Leone	SL7	West Africa	2019	11,475	3.88%
Senegal	SN7	West Africa	2018	6,186	2.93%
Togo	TG6	West Africa	2013/14	6,944	1.54%
Cameroon	CM7	Central Africa	2018/19	9,381	6.03%
DR Congo	CD8	Central Africa	2023/24	19,556	4.80%
Congo (Brazzaville)	CG6	Central Africa	2011/12	8,787	4.21%
Gabon	GA7	Central Africa	2019/20/21	6,916	14.73%
Chad	TD6	Central Africa	2014/15	18,452	0.67%

All sample reporting is based on DHS sampling weights.

The analytic sample was restricted to women who had completed higher education, defined using DHS standard coding. This category includes postsecondary, college, university, and higher professional training. Although the structure of tertiary education varies across countries, the DHS classification system ensures broad comparability across national contexts. After harmonization, a country identifier was generated and each country was assigned to one of four regional groupings: West Africa, East Africa, Southern Africa, or Central Africa. These groupings capture broad demographic and socioeconomic patterns that correlate with fertility behavior and educational opportunities. The groupings reflect macro-regional differences in marriage systems, economic development levels, and the pace of fertility transition, following standard regional classifications used in sub-Saharan African demographic research (22). However, grouping countries into broad regions may mask substantial within-region heterogeneity, as individual countries can vary considerably in fertility patterns, institutional contexts, and socioeconomic conditions (23). The regional estimates should therefore be interpreted as broad summaries rather than precise characterizations of individual countries. Table 1 provides detailed information on survey years, total women interviewed, and the proportion who attained higher education. The share of women with higher education varies widely across the region, ranging from 0.40 percent in Niger and 0.67 percent in Chad to 17.61 percent in Kenya and 16.10 percent in Lesotho. These differences highlight substantial inequalities in access to higher education across sub-Saharan Africa.

Missing data was minimal across key variables. Observations with missing values for age at first birth, wealth quintile, or covariates were excluded listwise. DHS does not provide imputed values for these variables, and no additional multiple imputation was performed.

### 2.2 Measures

Existing theoretical and empirical evidences recognized that age at first birth is one of the key proximate determinants of fertility (5, 24, 25). In this study, age at first birth is analysed as an outcome of independent demographic interest. While it is related to broader fertility patterns, the focus here is specifically on the timing of first birth among highly educated women.

The age of first birth information available in DHS birth history, and women who had not yet had a first birth were treated as censored at their age at interview. The event indicator therefore equals one for women who had a first birth and zero for those who remained childless at the time of interview. Time to event is measured in years. For women with a first birth, time corresponds to their reported age at first birth. For women without a first birth, time corresponds to their age at interview. Because DHS data are cross-sectional, time to first birth is reconstructed from retrospectively reported ages at first birth. This approach is standard in demographic research and allows for survival analysis even without prospective follow-up.

The main exposure is household wealth quintile. In the DHS, the wealth quintile is constructed using a principal components analysis (PCA) of household ownership of durable assets, housing characteristics, and access to basic services. The DHS wealth index construction is documented in (26). It is important to acknowledge that PCA is a data-reduction technique sensitive to the specific variables included and to the sample used. The DHS wealth index was constructed separately within each country using country-specific data, so quintile boundaries reflect relative within-country wealth rather than absolute cross-national living standards. What “rich” or “poor” means in assets and amenities differs across countries, and this should be kept in mind when interpreting pooled results (27). In this study, wealth is conceptualized as a multidimensional indicator of long-term economic status rather than short-term income, capturing structural economic advantage. Households are ranked by the resulting wealth index score and divided into five equal-sized quintiles from poorest to richest. The richest quintile refers to the top 20 percent of households in each country's wealth distribution.

Covariates included age (in years), birth cohort (year of birth), rural residence (rural vs. urban), media exposure (yes/no), and current employment status (working vs. not working). These variables capture key sociodemographic and informational factors that may influence the timing of first birth among highly educated women. Specifically, rural residence captures differential access to reproductive health services and labour markets (14); media exposure reflects access to information about reproductive health and modern family formation norms (25); and employment status proxies for opportunity costs of early childbearing and economic independence (15). Country fixed effects were included to account for unobserved national differences in fertility regimes, socioeconomic structures, and survey timing. Wealth quintile was entered as the main exposure, with the richest quintile serving as the reference category.

### 2.3 Survey design

All analyses incorporate the DHS complex sampling structure. Sampling weights were normalized, and the survey design was declared using primary sampling units and stratification variables. This ensures that all descriptive statistics, survival estimates, and regression models are representative of the national populations and that standard errors appropriately reflect the multistage sampling design.

### 2.4 Analytical strategy

The analysis proceeds in two stages. First, non-parametric Kaplan–Meier survival curves are used to describe the timing of first birth across wealth groups and regions. These curves provide an intuitive visualization of differences in the pace of entry into motherhood and allow for a descriptive assessment of socioeconomic and regional gradients. Second, the association between wealth and the hazard of first birth is estimated using proportional hazards models. The primary specification is the Cox proportional hazards model, which does not impose a functional form on the baseline hazard. The Cox model estimates the hazard of first birth for woman *i* at time *t* as:

$$\begin{array}{rl} h_{i}(t)= & \;h_{0}(t)\exp(\beta_{1}\mathrm{Wealt}h_{i}+\beta_{2}\mathrm{Rural}\;\mathrm{Residenc}e_{i}\\ & +\beta_{3}\mathrm{Birth}\;\mathrm{Cohor}t_{i}+\beta_{4}\mathrm{Media}\;\mathrm{Exposur}e_{i}\\ & \;+\;\beta_{5}\mathrm{Employment}\;\mathrm{Statu}s_{i}+\gamma_{c}\mathrm{Countr}y_{i}). \end{array}$$

Here, $h_{0}(t)$ is the unspecified baseline hazard, and the coefficients represent the log-hazard ratios associated with each covariate. The Cox model is estimated using survey-adjusted procedures to ensure correct inference. To assess the robustness of the findings, two parametric proportional hazards models were estimated: the Weibull and Gompertz models. These models impose specific shapes on the baseline hazard. The Weibull model assumes a baseline hazard of the form:

$$h_{0}(t)=\lambda pt^{\;p-1},$$

while the Gompertz model assumes an exponentially increasing or decreasing baseline hazard:

$$h_{0}(t)=\lambda e^{\gamma t}.$$

Both models estimate the same covariate effects as the Cox model but under different assumptions about how the hazard changes with age. Consistency across the Cox, Weibull, and Gompertz models strengthens confidence that the observed wealth gradient is not an artifact of baseline hazard specification.

## 3 Results

### 3.1 Descriptive characteristics of highly educated women

The analytic sample of 17,336 highly educated women is drawn from all four major subregions of sub-Saharan Africa, with the largest shares residing in West Africa (35.1 percent) and East Africa (30.1 percent), followed by Southern Africa (20.1 percent) and Central Africa (14.7 percent). Reflecting their advantaged socioeconomic position, 70.1 percent fall within the richest wealth quintile and 78.0 percent live in urban areas. Most respondents are currently employed, with 72.3 percent reporting paid work, and 39.9 percent report regular media exposure. The average age of women in the sample is 33.5 years, and the mean birth cohort is 1987. Full sample characteristics are reported in Table 2.

**Table 2:** Descriptive characteristics of highly educated women, demographic and health survey (*N* = 17,336).

Variable	Sample	Percentage
Region		
West Africa	6,085	35.1
East Africa	5,218	30.1
Southern Africa	3,485	20.1
Central Africa	2,548	14.7
Wealth quintile		
Poorest	139	0.8
Poorer	451	2.6
Middle	1,179	6.8
Richer	3,415	19.7
Richest	12,153	70.1
Residence		
Urban	13,522	78.0
Rural	3,814	22.0
Currently working		
No	4,802	27.7
Yes	12,534	72.3
Media exposure		
No	10,419	60.1
Yes	6,917	39.9
**Continuous Variables**		**Mean (Std. Error)**
Age of women (years)	17,336	33.52 (0.080)
Birth cohort (year)	17,336	1,987.05 (0.091)

All estimations are based on weighted sample.

### 3.2 Distribution of Age at first birth

**Figure 1:**
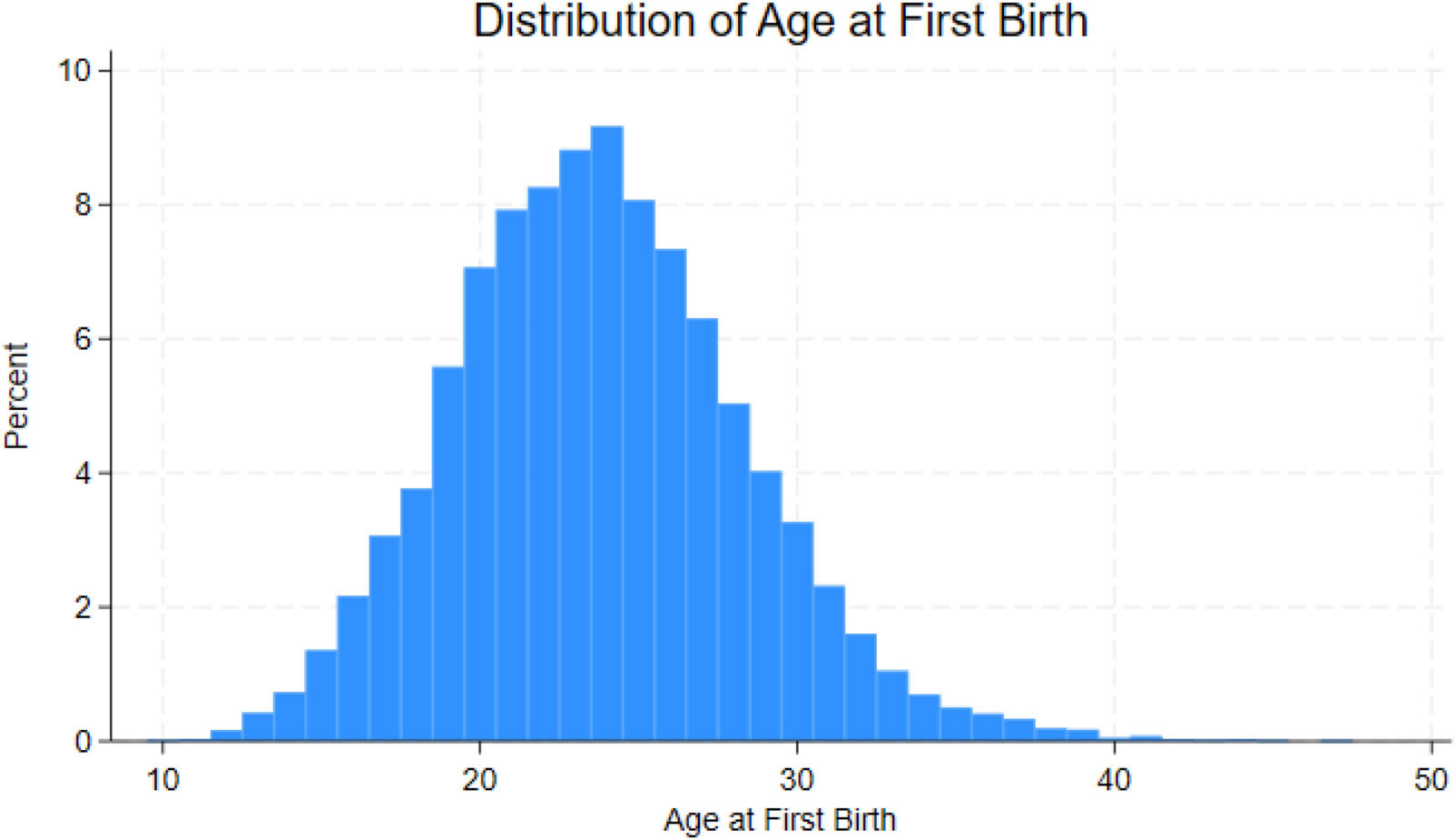
Histogram of Age at first birth among highly educated women.

The distribution of age at first birth is strongly concentrated in the early to mid-twenties, with a clear peak around age 24. The pattern is right-skewed, indicating that while most highly educated women have their first birth in their twenties, a smaller share delay childbearing into their thirties and beyond. This distribution is illustrated in Figure 1.

### 3.3 Wealth differences in the timing of first birth

**Figure 2:**
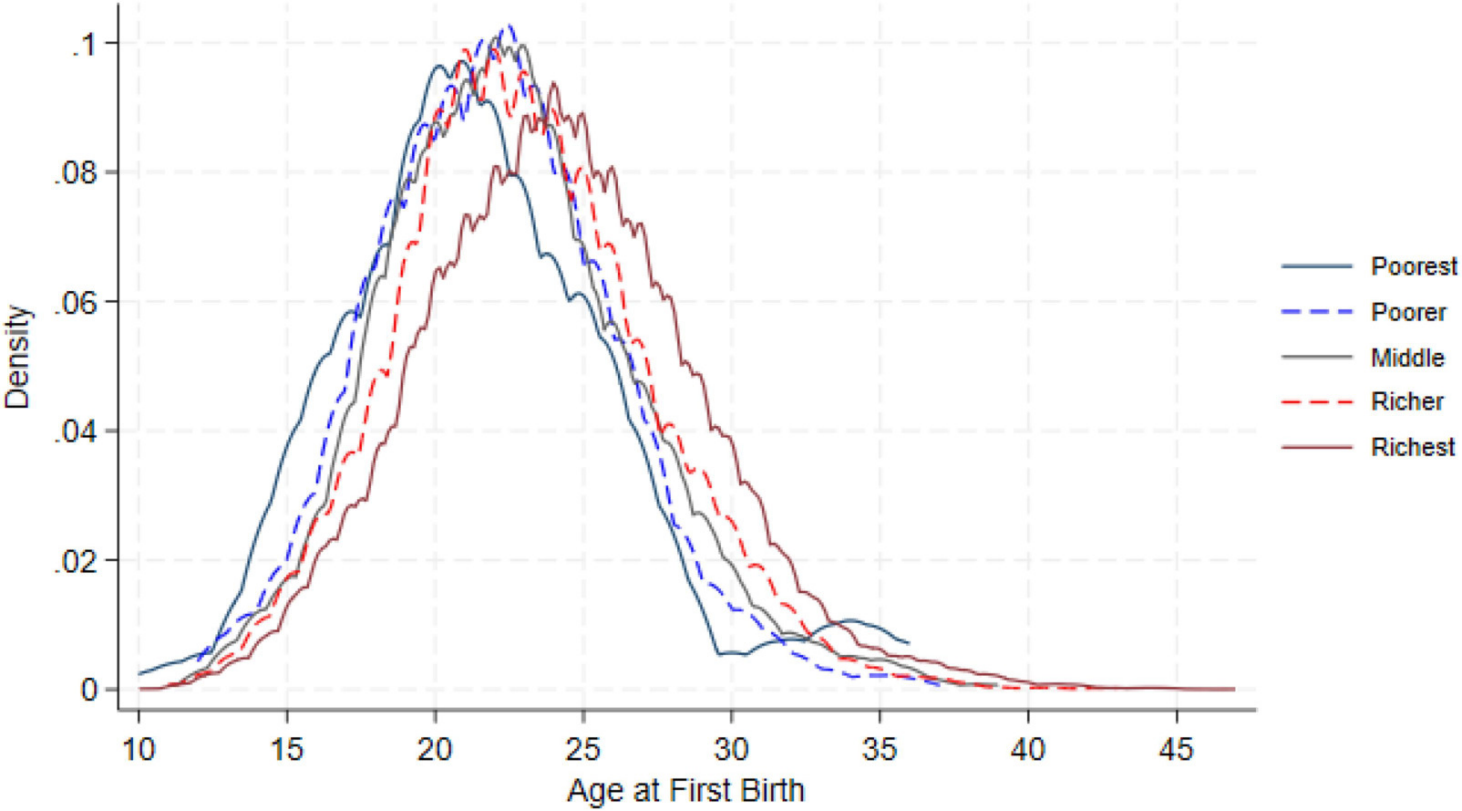
Weighted density of age at first birth by wealth quintile.

**Figure 3:**
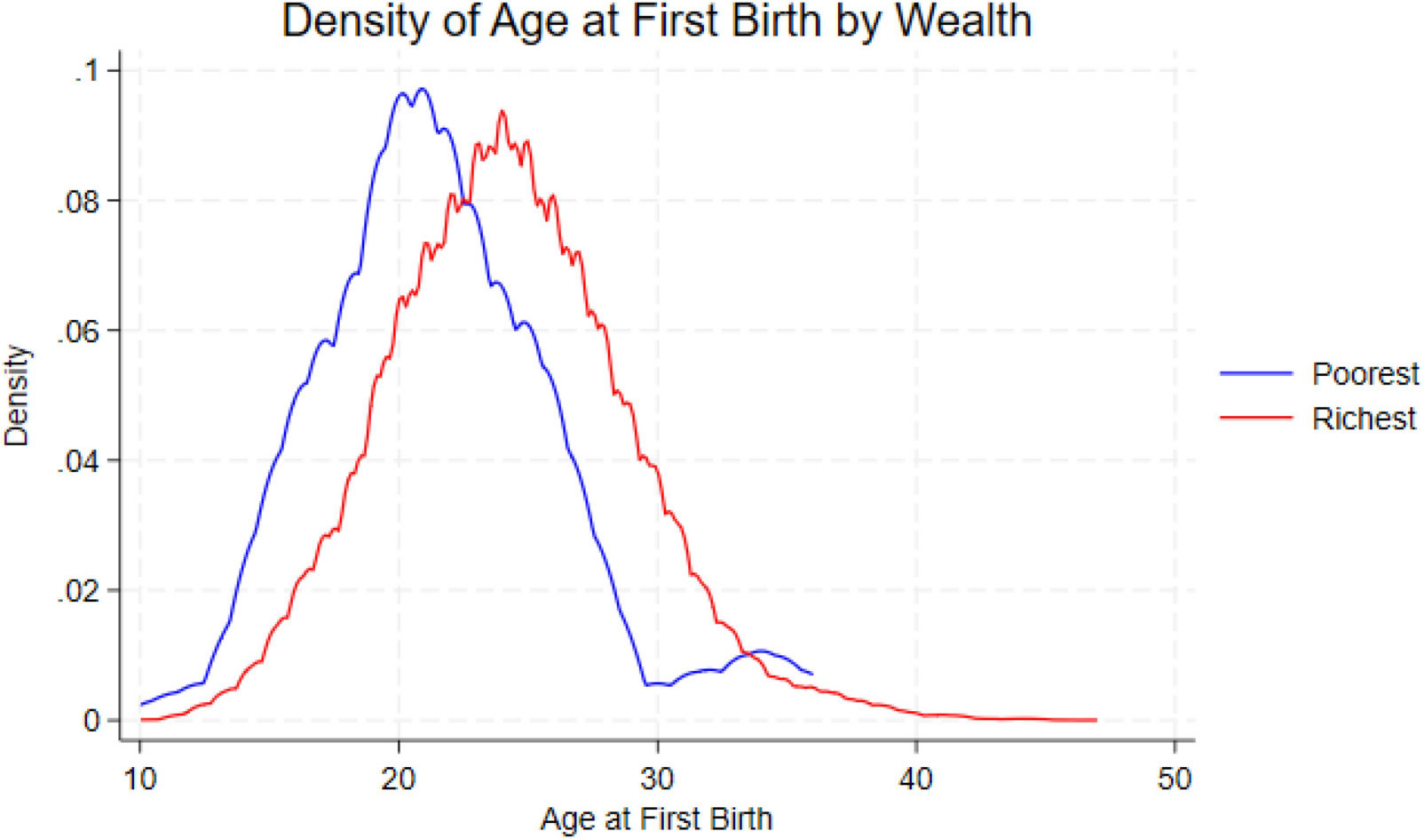
Density of age at first birth for poorest and richest women.

The timing of first birth changes clearly across wealth groups. Women in the poorest and poorer quintiles tend to have their first child earlier, with most first births happening around ages 19 to 21. As wealth increases, the peak of the distribution shifts to later ages. Women in the richer and richest quintiles show the latest timing, with first births most common between ages 23 and 25. When comparing only the poorest and richest groups, the difference becomes even more visible: the poorest women start childbearing several years earlier, while the richest women delay it until their mid-twenties. These patterns are shown in Figures 2, 3.

### 3.4 Kaplan–Meier estimates of first birth timing by household wealth quintile

**Figure 4:**
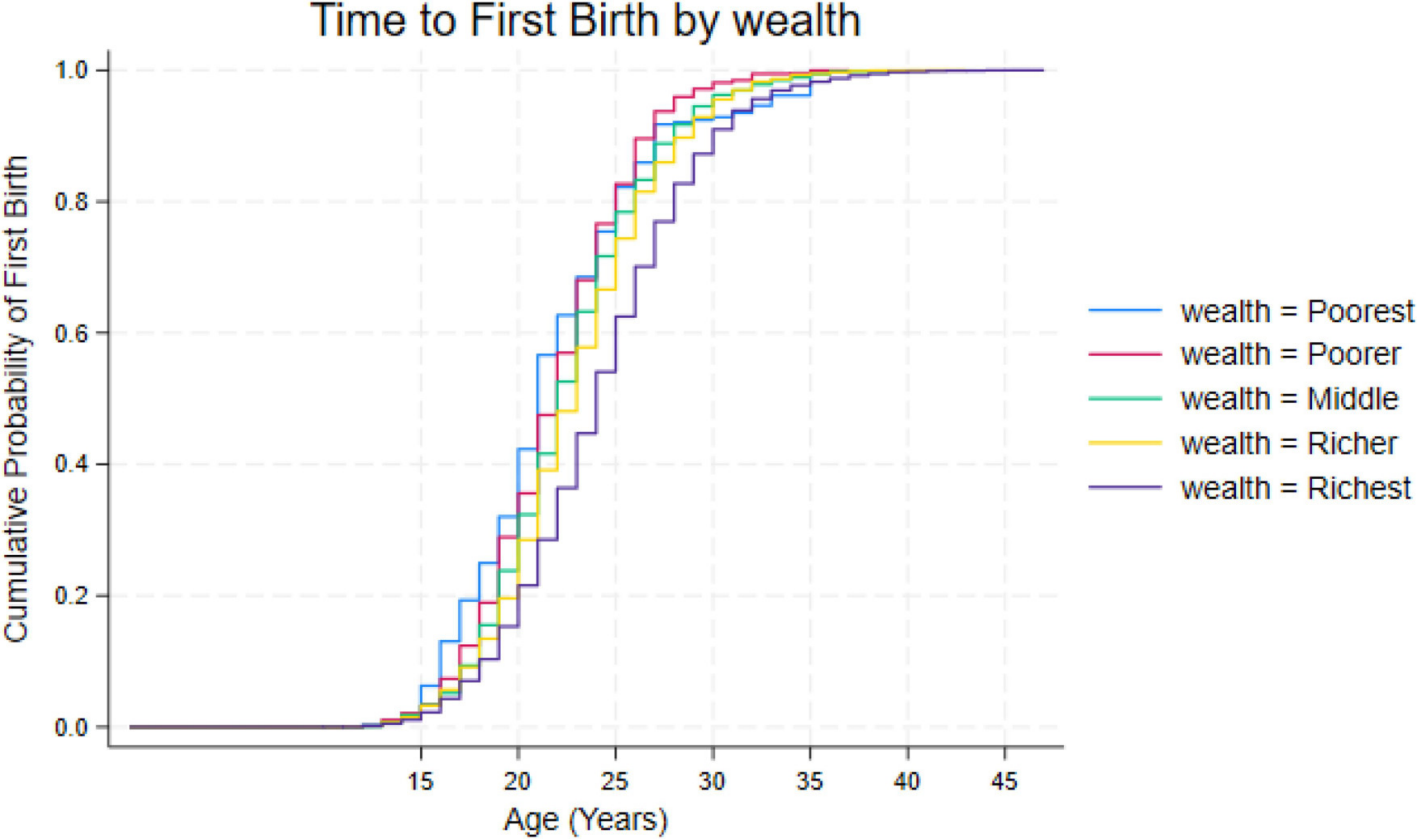
Kaplan–Meier survival curves for age at first birth by wealth quintile.

The Kaplan–Meier curves show clear differences in the timing of first birth across wealth groups. Women in the poorest quintile begin childbearing the earliest, with the curve rising steeply, with more than half having had a first birth by about age 22. The middle wealth groups transition more slowly, with the curve reaching the halfway point closer to age 24. The richest women show the slowest entry into motherhood, with the curve rising gradually through the early twenties and these wealth differentials in survival are displayed in Figure 4.

### 3.5 Regional patterns in first birth timing

**Figure 5:**
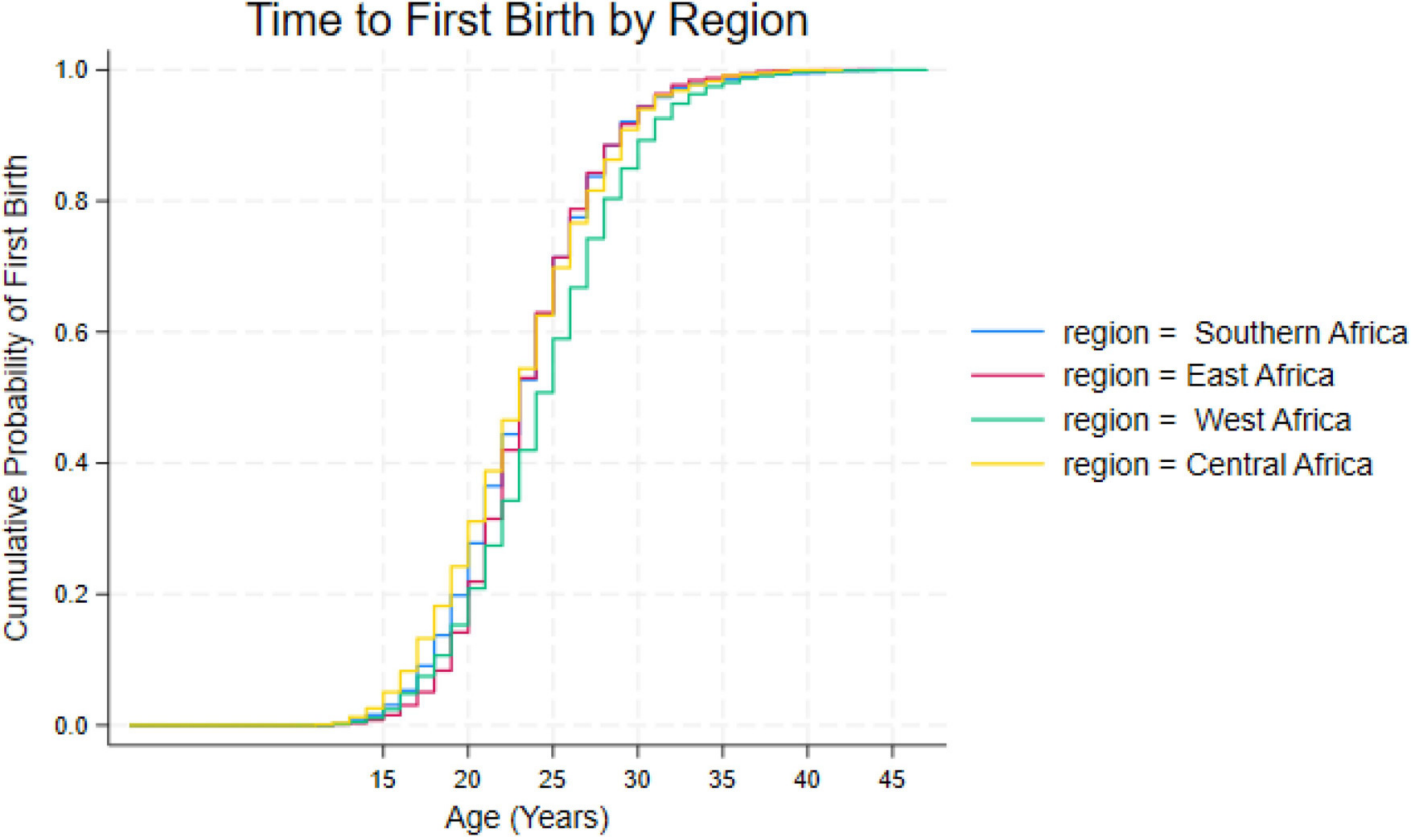
Kaplan–Meier survival curves for age at first birth by region.

The Kaplan–Meier curves show clear regional differences in the timing of first birth. Women in West and Central Africa begin childbearing the earliest, with their curves rising steeply between ages 18 and 22 and reaching the halfway point around age 23. East Africa shows a slightly later pattern, with the curve rising more gradually and the midpoint occurring closer to age 24. Southern Africa has the slowest transition into motherhood, with the curve rising slowly through the early twenties and regional survival curves are presented in Figure 5.

### 3.6 Household wealth gradients within regions

**Figure 6:**
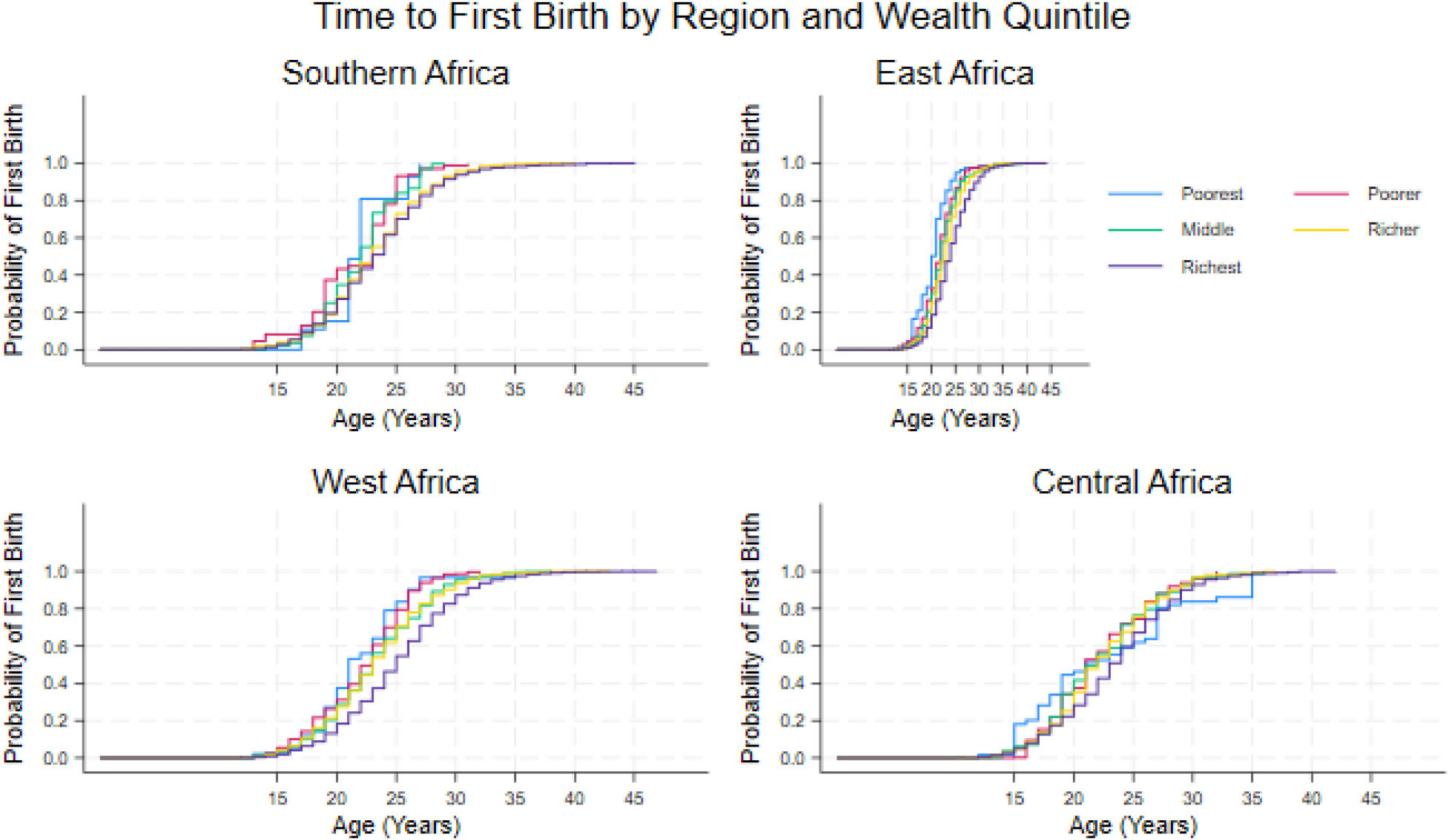
Household wealth quintile differentials in entering motherhood across selected regions.

Across all four regions, the Kaplan–Meier curves show a similar pattern: poorer women begin childbearing earlier, and richer women delay first birth. In West and Central Africa, the poorest groups experience the fastest transition into motherhood, with many women having their first birth between ages 18 and 22, while the richest groups reach this stage closer to ages 24 or 25. East Africa shows a slightly later pattern overall, but the wealth gradient remains clear, with richer women delaying first birth by several years. Southern Africa shows the slowest timing across all wealth groups, with even the poorest women reaching the midpoint of first births around age 24. Wealth differentials within each region are displayed in Figure 6.

### 3.7 Association between household wealth quintile and time to first birth among higher-educated women

The results show a strong and consistent wealth gradient in the timing of first birth. Compared with the richest women, those in the richer group have about a 19% higher hazard of having a first birth (HR = 1.194, SE = 0.028), women in the middle group have a 28% higher hazard (HR = 1.281, SE = 0.047), and those in the poorer group have a 42% higher hazard (HR = 1.420, SE = 0.078). Even the poorest group shows a 33% higher hazard (HR = 1.327, SE = 0.178), although the estimate is less precise. These patterns show that as wealth decreases, the likelihood of transitioning to first birth at any given age is higher. Estimates from the Weibull and Gompertz models are similar in both direction and magnitude, indicating that the observed wealth gradient is robust to alternative assumptions about the baseline hazard.

Among the other covariates, age is associated with a 7% lower hazard of first birth (HR = 0.928, SE = 0.030), meaning older women in the sample have a lower likelihood of transitioning into motherhood at a given time. Birth cohort shows no meaningful association. Rural residence is associated with a higher hazard by about 4% (HR = 1.038, SE = 0.024), while media exposure shows no clear association. Employment has a very small positive association, with a higher hazard by about 2% (HR = 1.017, SE = 0.022). The country fixed effects show large differences across settings. Several countries have much lower hazards than the reference (e.g., Ghana: 40% lower, Senegal: 54% lower, Côte d'Ivoire: 30% lower), while others show higher hazards, such as Liberia (64% higher) and Sierra Leone (24% higher). These results highlight that both socioeconomic status and national context are strongly associated with the timing of first birth among highly educated women. Full model results are reported in Table 3.

**Table 3:** Association between wealth and time to first birth among higher educated women.

Variable	Cox PH	Weibull PH	Gompertz PH
	HR (SE)	HR (SE)	HR (SE)
Wealth quintile (ref = Richest)			
Richer	1.194*** (0.028)	1.224*** (0.034)	1.233*** (0.036)
Middle	1.281*** (0.047)	1.308*** (0.056)	1.302*** (0.059)
Poorer	1.420*** (0.078)	1.467*** (0.091)	1.452*** (0.091)
Poorest	1.327** (0.178)	1.302* (0.203)	1.258 (0.194)
Other covariates			
Age	0.928*** (0.030)	0.912** (0.037)	0.901** (0.045)
Birth cohort	0.984 (0.032)	0.984 (0.040)	0.980 (0.049)
Rural residence	1.038* (0.024)	1.046 (0.029)	1.054* (0.033)
Media exposure	0.985 (0.018)	0.988 (0.023)	0.994 (0.027)
Employment	1.017 (0.022)	1.041 (0.027)	1.062** (0.032)
Country fixed effects			
Burkina Faso	0.623*** (0.074)	0.624*** (0.088)	0.656*** (0.105)
Benin	0.600*** (0.117)	0.578** (0.138)	0.575**(0.159)
Burundi	0.579** (0.134)	0.583* (0.166)	0.597 (0.200)
DR Congo	0.920 (0.082)	0.946 (0.103)	1.002 (0.134)
Congo (Brazzaville)	0.918 (0.370)	0.947 (0.466)	0.934 (0.540)
Côte d’Ivoire	0.698*** (0.080)	0.681*** (0.093)	0.695** (0.108)
Cameroon	0.749 (0.138)	0.753 (0.167)	0.769 (0.195)
Ethiopia	0.804 (0.323)	0.824 (0.406)	0.816 (0.472)
Gabon	0.867 (0.121)	0.841 (0.133)	0.827 (0.139)
Ghana	0.595*** (0.053)	0.575*** (0.063)	0.575*** (0.080)
Gambia	0.910 (0.139)	0.960 (0.173)	1.007 (0.201)
Guinea	1.061 (0.210)	1.085 (0.256)	1.087 (0.288)
Kenya	0.933 (0.081)	0.961 (0.099)	0.989 (0.121)
Comoros	0.424 ** (0.156)	0.397** (0.180)	0.384* (0.207)
Liberia	1.635*** (0.308)	1.767*** (0.372)	1.763** (0.392)
Lesotho	0.880 (0.080)	0.883 (0.102)	0.900 (0.134)
Mali	1.014 (0.103)	1.014 (0.126)	1.025 (0.156)
Mauritania	0.706*** (0.093)	0.680** (0.110)	0.659** (0.131)
Malawi	1.052 (0.104)	1.102 (0.134)	1.168 (0.176)
Mozambique	1.156 (0.143)	1.153 (0.182)	1.079 (0.248)
Nigeria	0.775*** (0.066)	0.769** (0.083)	0.785* (0.111)
Niger	0.625 (0.232)	0.653 (0.299)	0.679 (0.367)
Namibia	0.783 (0.264)	0.781 (0.323)	0.764 (0.372)
Rwanda	0.599*** (0.080)	0.588*** (0.095)	0.604*** (0.114)
Sierra Leone	1.239 (0.201)	1.280 (0.246)	1.287 (0.278)
Senegal	0.457*** (0.091)	0.425*** (0.103)	0.425*** (0.118)
Chad	1.083 (0.349)	1.108 (0.435)	1.090 (0.492)
Togo	0.492** (0.164)	0.472* (0.193)	0.473 (0.227)
Tanzania	0.655*** (0.071)	0.648*** (0.087)	0.677** (0.105)
Uganda	0.968 (0.235)	0.999 (0.295)	0.983 (0.338)
South Africa	0.860 (0.211)	0.871 (0.261)	0.865 (0.302)
Zambia	1.061 (0.104)	1.050 (0.132)	1.049 (0.173)
Zimbabwe	0.908 (0.381)	0.959 (0.498)	0.958 (0.591)

* *p* < 0.10.

** *p* < 0.05.

*** *p* < 0.01.

Significance levels.

Standard errors in parentheses. All models are survey-adjusted using DHS weights, clustering, and stratification.

## 4 Discussion

This study demonstrates that household wealth continues to shape the timing of first birth among highly educated women in sub-Saharan Africa, even after accounting for education, union formation, and national context. The findings challenge the assumption that education alone equalizes reproductive trajectories and instead show that economic stratification is independently associated with systematic differences in fertility timing within educated populations. It is important to note that fertility change is a multidimensional process shaped by many interacting factors, and education is best understood as one important contributor among others. The present study does not claim that education is the sole predictor of fertility decline, but rather that wealth exerts an independent association alongside education.

Across all model specifications, a clear and monotonic wealth gradient emerges. Compared with women in the poorest quintile, progressively wealthier women experience increasingly delayed transitions to motherhood, with the richest women exhibiting the slowest entry into first birth. The magnitude and consistency of this gradient across Cox, Weibull, and Gompertz models underscore that the association is robust and not driven by assumptions about the baseline hazard. These results align with emerging evidence of growing inequality in family formation across sub-Saharan Africa (12, 28), but extend this literature by showing that such stratification persists even among women who have attained higher education.

The study also clarifies the correlates through which wealth is associated with fertility timing among highly educated women. Wealthier women tend to face higher opportunity costs of early childbearing, reflecting stronger labour market attachment, greater access to high-quality employment, and higher expected returns to uninterrupted careers. They may also have greater access to effective contraception, higher-quality healthcare, and social networks that normalize delayed family formation (29). These correlates are observed independently of formal educational attainment and are consistent with why wealth remains a strong correlate of fertility timing even within the top subgroup. However, given the cross-sectional design of this study, these should be understood as plausible associational mechanisms rather than established causal pathways. The present analysis cannot confirm directionality, and future longitudinal or experimental work would be needed to establish causal claims.

The findings further extend the diverging destinies framework by demonstrating that socioeconomic stratification in family formation persists within highly educated populations. Rather than education being associated with convergence in reproductive behaviour, wealth continues to differentiate the timing of motherhood among women with similar levels of schooling (30). This suggests that economic resources remain a ke*y* axis of advantage, even among those who have already achieved high levels of human capital.

Other covariates behave as expected and help contextualize the wealth gradient. Later age at first union is the strongest predictor of delayed first birth, highlighting the central role of partnership formation in structuring fertility timing. Additional years of schooling are associated with later motherhood even within this highly educated sample, while being married or in a union is associated with a higher likelihood of transitioning to first birth (31). These results confirm that wealth differentials persist net of the most proximate determinants of fertility, reinforcing the conclusion that economic resources exert an independent influence.

Regional patterns provide further insight into how broader socioeconomic contexts are associated with these dynamics. Southern Africa exhibits the steepest wealth gradients in first birth timing, a pattern consistent with higher levels of economic inequality, more formalized labour markets, and greater returns associated with delaying childbearing among wealthier women. In contrast, East Africa shows earlier overall transitions to motherhood and more modest wealth differentials, while West Africa displays relatively compressed wealth gradients alongside later fertility timing. These patterns suggest that the association between wealth and fertility timing varies across regional contexts, including inequality levels, marriage systems, and labour market structures (9, 15).

Several factors may be correlated with the observed wealth gradient. Wealth tends to coincide with higher opportunity costs of childbearing, more effective fertility control through access to reproductive health services, and different patterns of partner selection and shared fertility preferences within unions. At the same time, recent evidence that short-term wealth increases are associated with higher fertility in some African contexts (32) cautions against simple economic interpretations. Long-term socioeconomic status, rather than temporary income changes, is more relevant for understanding fertility postponement among educated women.

The implications of these results are significant for understanding fertility change in sub-Saharan Africa. As educational attainment continues to expand, fertility decline may increasingly be associated with women who also possess substantial economic resources. In other words, as more women gain access to higher education, the pace and extent of fertility decline will likely be associated with whether those women also have the economic resources that make delayed childbearing feasible and preferable. However, the persistence of wealth-based differentials among educated women cautions against assuming uniform fertility responses to educational expansion alone. Rising economic inequality may instead be associated with divergent fertility regimes within educated populations, reinforcing patterns of “diverging destinies” in family formation (33, 34). Such stratification has important consequences for population dynamics and health inequalities It is also worth acknowledging that the regional groupings used in this study may mask important heterogeneity within and among countries. Countries in the same region can differ substantially in their fertility levels, institutional contexts, and wealth distributions, and the regional patterns reported here should be read as broad tendencies rather than uniform country-level characteristics., particularly given evidence that the benefits of maternal education for child health are strongest when combined with household wealth (35, 36). These interactions between education and wealth in shaping health and demographic outcomes have been further documented across sub-Saharan Africa (35–37).

Situating these findings within a broader global perspective helps clarify both their distinctiveness and their commonality. Evidence from other regions that experienced rapid fertility transitions reveals comparable patterns of socioeconomic differentiation in family formation (38). In Latin America, fertility decline accelerated from the 1960s,coinciding with urbanization, rising female labor force participation, and expanded access to contraception, yet disparities in the timing of first births across wealth groups persisted even as overall fertility levels converge (37, 39). Similar dynamics are observed in East and Southeast Asia, where fertility transitions were rapid but uneven, with more educated and wealthier women postponing childbearing to a greater extent than their less advantaged counterparts (40, 41). In Europe and the United States, this pattern has been conceptualized within the “diverging destinies” framework, which highlights widening inequalities in family formation between socioeconomically advantaged and disadvantaged groups (12).

While sub-Saharan Africa differs in important respects, including higher overall fertility, different marriage systems, and more limited social protection, existing evidence points to similar underlying patterns. Studies consistently show that both education and household wealth are associated with reproductive behavior, including age at first birth, in ways that likely reflect aspirations, access to resources, and exposure to modern reproductive norms (9, 14, 22). However, compared to other regions, fertility decline in sub-Saharan Africa remains more heterogeneous and stratified, with persistent inequalities in early childbearing and maternal outcomes across socioeconomic groups (15, 42). In this context, the present finding, that a wealth gradient in first-birth timing persists even among highly educated women, aligns with a broader global pattern in which economic resources continue to correlate with reproductive trajectories beyond formal schooling. As the fertility transition deepens and educational attainment expands across the region, such socioeconomic stratification in family formation is likely to become more pronounced, mirroring trends previously observed in other parts of the world.

## 5 Limitations

The use of the DHS limits the kind of analysis and inferences that can be made from our findings. Due to the cross-sectional nature of the DHS, we were unable to establish causality between the factors associated with the timing of first birth among highly educated women. Also, as a study from a secondary dataset, we are unable to assess the association between other residual variables such as the influence of culture, among others. The data was self-reported. As such, there is the possibility of social desirability bias and recall bias. Notwithstanding these limitations, we have a strong conviction that the use of a nationally representative dataset ensures that our findings are generalizable to the larger population. We applied robust analysis in this study which adds to the validity of the study findings. Also, this study is arguably the first to examine the socioeconomic determinants of first birth timing among highly educated women from a regional perspective across sub-Saharan Africa.

## 6 Policy implications

In low-resource settings, realistic policy levers include expanding social protection programmes that reduce the economic vulnerability associated with early childbearing, improving access to affordable and high-quality childcare, and strengthening employment opportunities for young women entering the labour market. These interventions can reduce the opportunity costs of delaying motherhood and support women in aligning their fertility preferences with their economic aspirations. Ensuring equitable access to comprehensive reproductive health services, including modern contraception and high-quality maternal healthcare, is also essential for enabling women across the wealth spectrum to exercise reproductive autonomy. Investments in these areas are particularly important in contexts where wealthier women have greater access to private healthcare and more effective fertility control.

Policies that reduce structural wealth inequality, such as targeted cash transfers, youth employment initiatives, and expanded social insurance systems, can help narrow the socioeconomic gaps that shape family formation. As educational attainment continues to rise across sub-Saharan Africa, addressing persistent economic disparities will be critical for ensuring that fertility transitions are both sustained and inclusive.

## 7 Conclusion

This study examined the association between household wealth and the timing of first birth among highly educated women in sub-Saharan Africa using pooled data from the most recent Demographic and Health Surveys across 34 countries. Applying survey-adjusted Kaplan–Meier survival curves and proportional hazards models (Cox, Weibull, and Gompertz), the analysis reveals a clear and persistent socioeconomic gradient in the transition to motherhood. Even among women who have attained higher education, household wealth remains a significant predictor of fertility timing. By age 25, more than half of women in the poorest wealth quintile had already experienced a first birth, compared to just over two-thirds of those in the richest quintile. Multivariate results consistently show that women in lower wealth categories face substantially higher hazards of first birth relative to the richest group, with the gradient remaining robust across different model specifications.

The findings demonstrate that socioeconomic stratification in family formation extends beyond educational attainment. While higher education is widely recognized as a powerful driver of fertility decline,economic resources continue to correlate with reproductive trajectories among highly educated women, including factors such as opportunity costs, access to quality employment, and differential exposure to norms supporting delayed childbearing. Regional variations further highlight that the strength of this wealth gradient is most pronounced in Southern Africa and relatively compressed in West and Central Africa.

By isolating the independent effect of household wealth within a highly educated subpopulation, this study extends the “diverging destinies” framework to the sub-Saharan African context. It demonstrates that educational expansion alone is insufficient to eliminate socioeconomic differentials in reproductive timing. As female educational attainment continues to increase across the region, policies aimed at reducing structural wealth inequalities, through social protection programmes, economic empowerment initiatives, and equitable access to quality reproductive health services, will be critical for fostering more equitable fertility transitions and supporting broader goals of sustainable development.

## Data Availability

Publicly available datasets were analyzed in this study. This data can be found here: This study is a secondary analysis of publicly available, de-identified data from the Demographic and Health Surveys (DHS) program. Ethical approval for each DHS survey was obtained by ICF International and its implementing partners from the institutional review boards in the United States (at ICF) and the respective country. All participants provided informed consent. No separate ethical approval was required for this analysis.
